# Impacts of insecticide treated bed nets on *Anopheles gambiae* s.l. populations in Mbita district and Suba district, Western Kenya

**DOI:** 10.1186/1756-3305-7-63

**Published:** 2014-02-11

**Authors:** Kyoko Futami, Gabriel O Dida, George O Sonye, Peter A Lutiali, Mercy S Mwania, Scholastica Wagalla, Jecinta Lumumba, James O Kongere, Sammy M Njenga, Noboru Minakawa

**Affiliations:** 1 Department of Vector Ecology and Environment, Institute of Tropical Medicine, Nagasaki University, Nagasaki, Japan; 2 School of Public Health, Maseno University, Maseno, Kenya; 3 ASK Community Project, Mbita, Kenya; 4 NUITM-KEMRI Project, Kenya Medical Research Institute, Nairobi, Kenya; 5 Eastern and Southern Africa Centre of International Parasite Control (ESACIPAC), Kenya Medical Research Institute, Nairobi, Kenya

**Keywords:** *Anopheles gambiae*, *Anopheles arabiensis*, ITN, Species composition, Malaria

## Abstract

**Background:**

Abundance and species composition of sympatric malaria vector species are the important factors governing transmission intensity. A widespread insecticidal bed net coverage may replace endophagic species with exophagic species. However, unique local environments also influence a vector population. This study examined the impacts of insecticidal bed nets on *An. gambiae* s.l populations in Mbita District and Suba District.

**Methods:**

The species compositions of *An. gambiae* s.l. larvae were compared between 1997, 2009 and 2010 and between geographical areas. The abundance and species composition of *An. gambiae* s.l. females resting indoors were compared between 1999, 2008 and 2010 and between geographical areas. Bed net coverage was also examined temporally and spatially, and its relationships with vector abundance and species composition were examined.

**Results:**

The relative abundance of *An. gambiae* s.s. larvae was 31.4% in 1997, decreasing to 7.5% in 2008 and 0.3% in 2010. The density of indoor resting *An. gambiae* s.l. females decreased by nearly 95%, and the relative abundance of *An. gambiae* s.s. females decreased from 90.6% to 60.7% and 72.4% in 2008 and 2010, respectively. However, the species composition of indoor resting *An. gambiae* s.l. females changed little in the island villages, and *An. gambiae* s.s. remained dominant in the western part of the study area. The density of house resting females was negatively associated with the number of bed nets in a retrospective analysis, but the effect of bed nets on species composition was not significant in both retrospective and cross-sectional analyses.

**Conclusion:**

An increase in bed net coverage does not necessarily replace endophilic species with exophilic species. The effect of bed nets on *An. gambiae* s.l. populations varies spatially, and locally unique environments are likely to influence the species composition.

## Background

Understanding the relative abundance of sympatric malaria vector species is important for vector control, because each species has different vectorial capacity, which reflects on local malaria transmission intensity [1-3]. The most well-known sympatric malaria vectors are *Anopheles gambiae* s.s. Giles and *An. arabiensis* Patton, which belong to the *An. gambiae* complex group (hereafter *An. gambiae* s.l.). Both species inhabit small sun-lit pools [4,5], and often occur in the same geographical areas [6,7]. However, their adult feeding habits and resting sites differ; *An. gambiae* s.s. is more anthropophagic and endophilic than *An. arabiensis*[8-10]. Thus *An. gambiae* s.s. is considered the most efficient malaria vector in Africa; an area with abundant *An. gambiae* may have a high transmission risk. However, an area with abundant *An. arabiensis* does not necessarily have a lower transmission risk, as insecticide-treated bed nets (ITNs) and indoor residual spraying (IRS) are less effective against this exophilic species [11].

Their ecological differences imply that an introduction of ITNs or IRS alters the vector species composition. Recent studies have reported that the relative abundance of indoor resting *An. gambiae* s.s. fell compared with *An. arabiensis* following the introduction of ITNs in Kenya and Tanzania [3,12-16]. A change in predominant species was also observed following a vector control using IRS in western Kenya during the 1970s [17]. Nevertheless, the replacement of a predominant species should be confirmed in larvae, as sampling indoor resting vectors overestimates the abundance of *An. gambiae* s.s. On the other hand, ITNs and IRS may not have reduced the abundance of *An. gamibae* s.s. as much as recent studies have suggested, as its feeding place may have shifted outdoors to avoid insecticides [3,18,19].

Moreover, this phenomenon requires further careful interpretation, as an environmental change can also influence local mosquito species composition. *Anopheles arabiensis* often dominates in drier seasons and areas [7,20-22], thus the recent climate change or variability may have affected the composition of mosquito species. In addition, the effects of ITNs and IRS may vary spatially, as most environmental factors, (e.g. land use, local climate and livestock distribution), are spatially heterogeneous.

The main objective of this study was to reveal if an introduction of ITNs had altered *An. gambiae* s.l. populations in Mbita District and Suba District in the Nyanza Province of western Kenya. In particular, we wanted to confirm if ITNs had reduced densities of the indoor resting malaria vectors and altered their species composition at both adult and larval stages, and to reveal if the effects of ITNs on their density and abundance were geographically heterogeneous.

## Methods

### Study area

Mbita District and Suba District lie within the Kenyan part of the Lake Victoria basin. The study area included five inhabited islands, Kibuogi, Mfangano, Ngodhe, Rusinga and Takawiri. Both districts formed a single district (Suba District) prior to 2011, but the western region was later designated as Mbita District. The total area of both districts combined is approximately 1055 km^2^, however, the hilly regions were excluded from this study as malaria vectors are not abundant in these areas. The study area was mostly deforested by the mid-1990s with the exception of a few small patches of forest remaining in the hills, the vegetation cover had remained mostly unchanged since then. The rainfall pattern in the area is bimodal, with a long rainy season occurring from March through May, and a short rainy season occurring around November.

Most inhabitants belong to the Luo and Suba ethnic groups whose main socio-economic activities are traditional small-scale fishing and farming. Most houses are constructed of a stick framework, plastered with a mixture of mud and cow dung, commonly covered with a corrugated iron roof or, in few instances, with thatch. Kenya’s Ministry of Health, with assistance from the Global Fund, started a distribution of LLINs in this area in late 2006. However, malaria transmission remains high [23,24]. There had not been additional systematic malaria vector interventions in the study area since 1997.

The main malaria vectors in the study area are *An. gambiae* s.s., *An. arabiensis* and *An. funestus* s.s. Giles. A study in 1997 reported that 65.5% of *An. gambiae* s.l. larvae sampled in the study area were *An. arabiensis*, and the species composition was spatially heterogeneous [4,24,25]. However, in 1999 *An. gambiae* s.s. dominated indoors [25,26].

### Larval mosquitoes

In May 2009 (during the rainy season), we collected anopheline larvae in nine areas, Gembe, Luanda, Mbita, Nyandiwa, Ogongo, Owichi, Rusinga, Valley South, and Valley North, where a previous study surveyed in February and March, 1997 [4]. Larvae were also collected in January and February, 2010, as the species composition may change seasonally [20]. However, we considered the 1997 and 2010 samples as being from rainy periods, as both periods received considerable rainfall. We collected larvae at the same sites as the 1997 survey or at sites with similar habitats close to previous sites [4]. Larvae were collected using a 350 ml dipper (350 ml; BioQuip Products, Rancho Dominguez, USA), and collected larvae were immediately soaked in 100% ethanol. Geographical coordinates of each collection site were recorded using a GPS unit (GPSmap 60CSx, Garmin International Inc., Olathe, USA).

Collected larvae were identified into *An. gambiae* s.l. under a dissecting microscope using morphological keys [27]. DNAs of *An. gambiae* s.l. larva were extracted using the ethanol precipitation method. The DNA samples were identified into *An. gambiae* s.s. or *An. arabiensis* using the rDNA-polymerase chain reaction (PCR) method [28]. In cases where the initial PCR amplification failed, the PCR analysis was repeated up to two times. Samples not successfully amplified were scored as unknown. Approximately 100 randomly selected larvae were identified into species for each area when a sample size exceeded 100.

### Indoor resting female mosquitoes

Indoor resting anopheline females were collected from 257 houses in 19 villages during May 2008, and from 470 houses in 31 villages during May 2010 (Table 1). Both sampling periods were within a rainy period. Each survey included the same 144 houses in the 11 villages as the 1999 survey to allow for comparison [29]. When the same houses were not available, the nearest houses of a similar size and construction were selected. Some houses were visited two or three times when an insufficient number of mosquitoes was obtained during the first sampling session. The study area was expanded in 2008 and 2010 to cover the entire Mbita District in order to explore spatial variations of malaria vector abundance and species composition, as mosquito samples were available from other research projects [30]. Indoor resting females were collected using the pyrethrum spray catch method (PSC) [31]. The collected females were immediately placed in a cool box with ice, and preserved in a −20°C freezer for subsequent PCR identification. During adult mosquito collection, we asked the head of each house the number of bed nets, the number of residents, and what number of them had slept under nets the previous night.

**Table 1:** Mean mosquito densities in each year, region, island and mainland

**Factors**	***An. gambiae *s.l.**	***An. gambiae *s.s.**		***An. arabiensis***	
	**Mean (SD)**	**Mean (SD)**	**%**	**Mean (SD)**	**%**
11 villages					
* Year*					
1999	28.5 (42.79)	25.8 (41.61)	90.6	2.7 (4.42)	9.4
2008	2.0 (4.04)	1.3 (3.35)	60.7	0.8 (1.72)	39.3
2010	2.0 (3.49)	1.5 (2.84)	72.4	0.5 (1.42)	27.6
* Region*					
Western	6.1 (21.04)	5.2 (20.27)	86.6	0.8 (2.69)	13.4
Eastern	7.1 (20.69)	6.0 (19.71)	85.2	1.1 (2.26)	14.8
* Island/mainland*					
Island	5.0 (13.07)	4.0 (11.32)	80.5	1.0 (2.74)	19.5
Mainland	9.3 (29)	8.3 (28.54)	89.9	0.9 (1.91)	10.1
Total	6.7 (20.84)	5.7 (19.95)	85.7	1.0 (2.45)	14.3
19 villages					
* Year*					
2008	2.4 (4.99)	0.8 (2.65)	32.4	1.6 (3.88)	67.6
2010	2.6 (7.52)	1.3 (2.75)	54.1	1.4 (6.92)	45.9
* Region*					
Western	1.9 (3.71)	1.5 (3.36)	77.8	0.4 (1.2)	22.2
Central	3.5 (5.74)	1.7 (3.1)	44.3	1.8 (4.35)	55.7
Eastern	2.4 (7.88)	0.6 (1.94)	27.2	1.8 (7.54)	72.8
* Island/mainland*					
Island	2.2 (3.76)	1.6 (2.93)	72.1	0.6 (1.59)	27.9
Mainland	2.7 (7.76)	0.8 (2.53)	29.7	1.9 (7.21)	70.3
Total	2.5 (6.56)	1.1 (2.71)	43.9	1.5 (5.82)	56.1
31 villages (2010)					
* Region*					
Western	1.5 (2.98)	1.1 (2.47)	75.1	0.4 (1.27)	24.9
Central	3.2 (8.98)	1.5 (2.97)	53.9	1.7 (8.42)	46.1
Eastern	1.3 (2.41)	0.2 (0.45)	12.4	1.1 (2.23)	87.6
* Island/mainland*					
Island	2.2 (3.61)	1.7 (2.91)	74.5	0.5 (1.45)	25.5
Mainland	2.3 (7.58)	0.7 (2.12)	34.8	1.6 (7.2)	65.2
Total	2.2 (6.61)	1 (2.43)	47.9	1.3 (6.06)	52.1

### Statistical analyses

Maximum likelihood statistical models were used to reveal factors that affect the density of malaria vector species (*An. gambiae* s.l*.*, *An. gambiae* s.s*.* and *An. arabiensis*) and species composition within *An. gambiae* s.l. (the relative abundance of *An. arabiensis*) in the study area. A full model included all relevant covariates, this model was then simplified until the Akaike Information Criterion (AIC) was minimized following the stepwise removal of covariates. The significance (P > 0.05) of each fixed variable remaining in the optimal model was estimated using a log-likelihood ratio test. The Statistical Package R version 3.0 was used for all analyses [32].

A binomial generalized linear mixed model (GLMM) (employing the R package ‘lme4’) was used to compare the relative abundance of *An. arabiensis* larvae between 1997, 2009 and 2010 [33]. The year was assigned as a fixed factor, while area was considered as a random factor in the model [34].

For indoor resting females, we compared the average numbers of *An. gambiae s.l.*, *An. gambiae s.s.* and *An. arabiensis* from the 11 villages studied between 1999, 2008 and 2010 using a Poisson GLMM. Year was a fixed factor, while village and house were assigned as random factors. In the analyses, two types of geographical variables were also included to explore spatial heterogeneity. We categorized the villages into “island” and “mainland” for the first geographical variable (Island/mainland). The second geographical variable was constructed by grouping the villages using a cluster analysis based on the geographical distance between each pair of villages (employing R package ‘stats’). In these analyses, we could not include the variables related to bed nets and residents, as they were not available for the 1999 data set. Furthermore, we compared the densities of each taxon between years for each village separately, assigning year as a fixed factor and house as a random factor, which would provide us a picture of how the temporal changes in density varied among villages (or spatially).

For adult species composition, we also used a binomial GLMM to compare the relative abundance of *An. arabiensis* between years, incorporating the same fixed and random factors used in the analyses of density. Then, we examined temporal change in species composition within each village separately. When no mosquitoes were amplified by PCR for a single house, the house was excluded from the analysis.

Using the adult mosquito data set from the 19 villages studied during 2008 and 2010, we revealed the factors affecting the density of each taxon and species composition. As the bed net coverage peaked in the area during 2008, two years after the mass bed net distribution campaign and then began to decline, we wanted to know if this decline affected vector density and species composition. We were also interested in the spatial variations of vector density and species composition, and their changes between 2008 and 2010. For the analyses, we used a Poisson GLMM for density and a binomial GLMM for species composition including house and village as random factors. Fixed factors assigned were year, the number of bed nets, the number of residents and the number of residents that had slept under nets on the previous night in each house, and the two geographical variables used in the analysis for the 11 villages. Furthermore, we examined the spatial variations of vector density and species composition of *An. gambiae* s.l. females with the data set from the larger area including 31 villages in 2010, applying the same statistical procedure mentioned above.

### Ethical consideration

This study was approved by the Ethical Committee of Kenya Medical Research Institute (KEMRI) (SSC No. 2131), Kenya. After the study was explained to the household heads in the local language, informed consent was obtained from them prior to sampling mosquitoes from their houses and interviewing them about bed net usage.

## Results

### Larval species composition in 1997, 2009 and 2010

**Figure 1:**
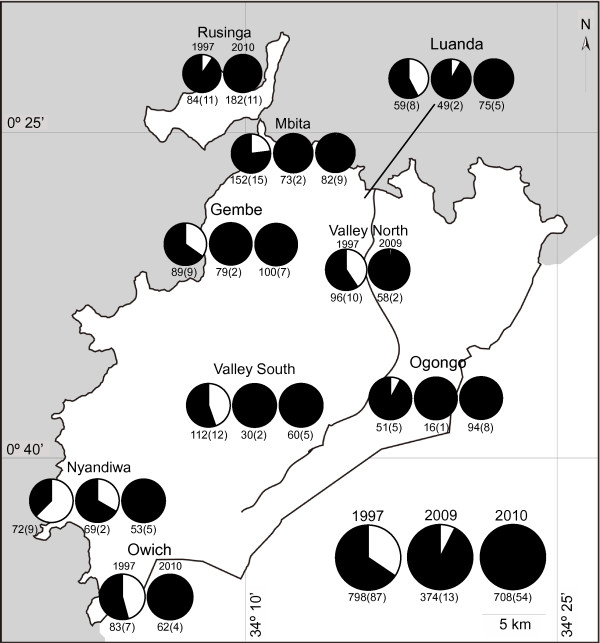
**Relative abundances of *An. gambiae *s.s. and *An. arabiensis *larvae in 1997, 2009 and 2010.** The white and black pies are proportions of *An. gambiae* s.s and *An. arabiensis,* respectively. The left, middle and right pie graphs are results of 1997, 2009 and 2010, respectively. A number below each pie graph is the number of collected larvae, and a number in parentheses is the number of habitats.

Since we could not collect enough larvae in Ruri, Owichi and Rusinga in 2009 and in Ruri and Valley North in 2010, the data from these areas were not included in the analysis of species composition. A total of 374 *An. gambiae* s.l. larvae were collected in 2009. Of these, 28 (7.5%) were *An. gambiae* s.s., and 346 (92.5%) were *An. arabiensis* (Figure 1), while the proportions of *An. gambiae* s.s*.* and *An. arabiensis* were 31.4% and 68.6% in 1997, respectively [4]. In 2010, the total number of *An. gambiae* s,l. larvae was 708, and the relative abundance of *An. gambiae* s.s. further decreased to 0.3%. The best binomial GLMM for the relative abundance of *An. arabiensis* larvae selected two fixed variables, year and area, with village as a random variable (Additional file 1: Table S1). While the effect of year was statistically significant (χ^2^ = 90.37, df = 2, P *<* 0.001), area was not significant (χ^2^ = 2.62, df = 1, P *<* 0.105).

### Adult species compositions of 11 villages in 1999, 2008 and 2010

In 1999, Chen *et al*. collected 4099 *An. gambiae* s.l. females from the 11 surveyed villages (Additional file 2: Table S2) [29]. Of these, 384 (9.4%) were *An. arabiensis*, and 3715 (90.6%) were *An. gambiae* s.s. In 2008, 556 *An. gambiae* s.l. females were collected from the same 11 villages; 468 of these were used for PCR identification, of which 440 (94.0%) were successfully amplified, identifying 173 *An. arabiensis* (39.3%), and 267 *An. gambiae* s.s. (60.7%). In 2010, 865 *An. gambiae* s.l. were collected, and 730 individuals were used for species identification. Of these, 188 (27.6%) were *An. arabiensis,* and 492 (72.4%) were *An. gambiae* s.s.

**Figure 2:**
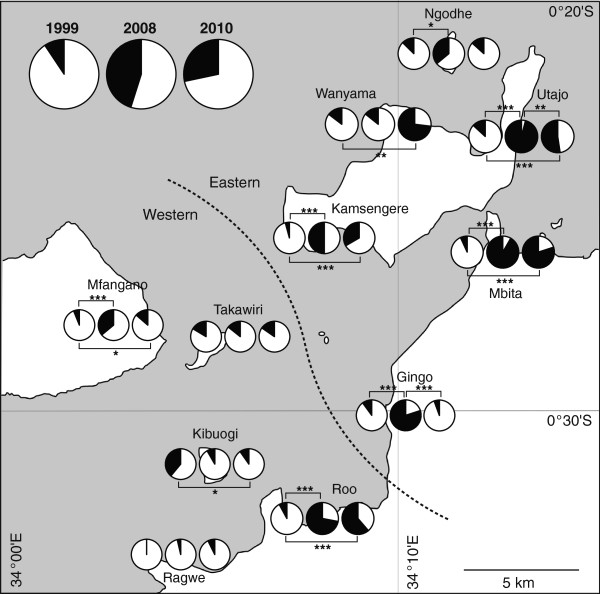
**Relative abundances of *An. gambiae *s.s. and *An. arabiensis *females in 1999, 2008 and 2010.** White and black pies are proportions of *An. gambiae* s.s and *An. arabiensis,* respectively. Left, middle and right pie graphs are results of 1999, 2008 and 2010, respectively. A number below each pie graph is the number of collected larvae, and a number in parentheses is the number of habitats. Stars show a level of significance (* < 0.05, ** < 0.01, *** < 0.001). A dotted line shows the border of two geographical regions.

A cluster analysis based on geographical distance grouped the 11 surveyed villages into two separate regions, the eastern region and the western region (Figure 2). The best model for the relative abundance of *An. arabiensis* females selected one fixed variable, year, and two random variables, house and village (Additional file 3: Table S3). The relative abundance significantly differed between the years (χ^2^ = 119.82, df = 2, P < 0.001). The relative abundances in 2008 and 2010 were significantly greater than in 1999, and those in 2010 were significantly lower than in 2008.

When the relative abundance of *An. arabiensis* was compared between the years for each village separately, the relative abundance did not differ significantly in Takawiri and Ragwe (Figure 2). Although the relative abundance of *An. arabiensis* in Wanyama had not significantly changed between 1999 and 2008, it increased significantly during 2010. On the other hand, the relative abundance of *An. arabiensis* significantly decreased in Kibuogi between 1999 and 2010, and decreased in Gingo and Utajo between 2008 and 2010. In the remaining villages, the relative abundance increased in 2008 when compared to 1999, but was stable or decreased during 2010.

### Adult densities of 11 villages in 1999, 2008 and 2010

The best Poisson GLMM for adult density selected only one fixed variable, year, for *An. gambiae* s.l. and *An. gambiae* s.s., and two random variables, house and village (Additional file 4: Table S4). The selected fixed variable was statistically significant (*An. gambiae* s.l.: *χ*^*2*^ = 940.78, df = 2, P < 0.001; *An. gambiae* s.s.: *χ*^*2*^ = 100.64, df = 2, P < 0.001). The density of *An. gambiae* s.l*.* reduced by 93% in 2008 and 2010 when compared with 1999, and the density of *An. gambiae* s.s. had reduced by 96% in 2008 and 95% in 2010 when compared with 1999 (Table 1). The best model for *An. arabiensis* selected two fixed variables, year and region, and the two random variables (Additional file 4: Table S4). However, the difference between the western region and eastern region was not statistically significant (*χ*^*2*^ = 2.88, df = 1, P = 0.090). The density of *An. arabiensis* reduced by 59% in 2008 and 70% in 2010 compared with 1999 (Table 1). The density of *An. arabiensis* in 2010 had reduced by 37% since 2008.

**Figure 3:**
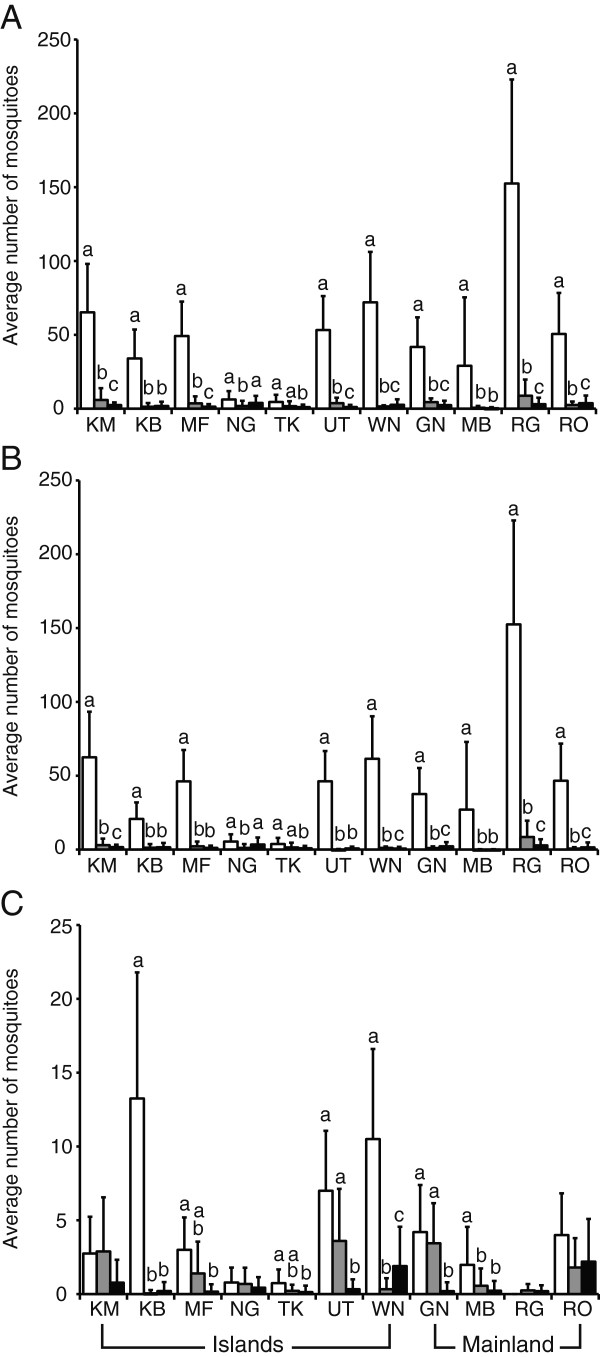
**Densities of *An. gambiae *s.l. (A), *An. gambiae *s.s. (B) and *An. arabiensis *(C) females in 1999, 2008 and 2010.** Different letters on error bar show significant difference by a multiple comparison test. White, gray and black bars are densities of 1999, 2008 and 2010, respectively.

When their densities were compared between the years for each village separately, *An. gambiae* s.l. numbers significantly declined between 1999 and 2008 in all villages except for Takawiri (Figure 3A). In 2010, the density of *An. gambiae* s.l. in most villages decreased compared with 2008 or was stable; however, an increase was seen in Ngodhe, Wanyama, and Roo. The density of *An. gambiae* s.s. showed a similar pattern to *An. gambiae* s.l. except Mfangano and Utajo. In these two villages, its density did not change significantly between 2008 and 2010 (Figure 3B). On the other hand, the density of *An. arabiensis* had a different pattern from the other two mosquito groups. Its density did not change significantly between the three years in Kamsengere, Ngodhe, Ragwe, and Roo (Figure 3C). A large decrease between 1999 and 2008 was observed only in Kibuogi, Wanyama and Mbita. The density increased in Wanyama in 2010, but it was stable in the other two locations. The remaining four villages had significantly lower densities in 2010 compared with 1999.

### Bed net coverage in 2008 and 2010

**Figure 4:**
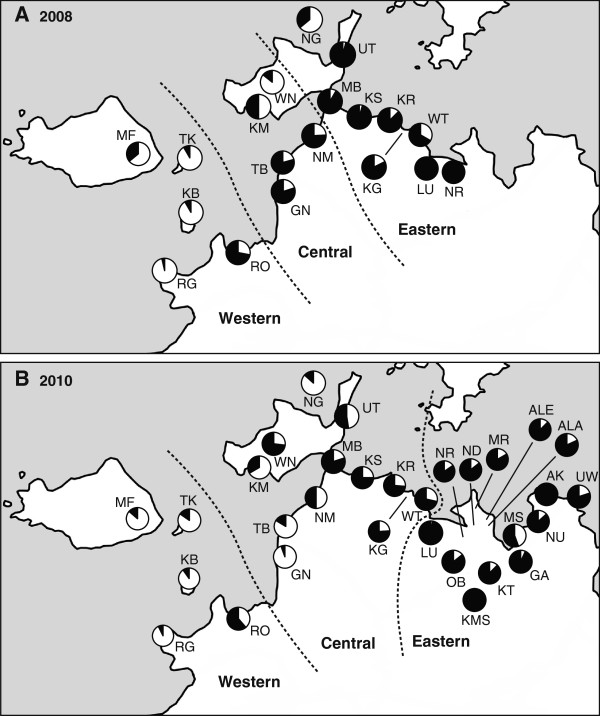
**Relative abundances of *An. gambiae *s.s. and *An. arabiensis *females in 2008 (A) and 2010 (B).** White and black pies are proportions of *An. gambiae* s.s and *An. arabiensis,* respectively. Dotted lines are the borders of geographical regions.

A cluster analysis grouped 19 villages into three geographical areas, western region, central region and eastern region (Figure 4A). The best Poisson GLMMs selected one fixed variable, year, and two random variables, house and village, for the numbers of bed nets, the number of residents and the number of residents having slept under bed nets in the 19 villages (Additional file 5: Table S5). The effect of year was statistically significant for the three dependent variables (bed net: *χ*^*2*^ = 145.05, df = 2, P < 0.001; resident: *χ*^*2*^ = 4.84, df = 1, P = 0.028; residents under nets: *χ*^*2*^ = 22.95, df = 1, P < 0.001). The model for the number of bed nets also selected island/mainland; however, the fixed variable was not statistically significant (*χ*^*2*^ = 2.06, df = 2, P = 0.152). For the numbers of residents and residents having slept under bed nets, the best models also selected geographical region; however, the fixed variable was not statistically significant in either case (resident: *χ*^*2*^ = 5.50, df = 2, P = 0.062; residents under nets: *χ*^*2*^ = 4.28, df = 2, P = 0.118). The number of bed nets per house decreased by approximately 48% between 2008 and 2010, the number of residents decreased by approximately 10% in 2010, and the number of residents having slept under bed nets also decreased by approximately 23% in 2010 (Table 2).

**Table 2:** Means of bed nets, residents and residents slept under net per house

**Factors**	**N**	**Bed net**	**Residents**	**Under Net**
2008 (19 villages)				
* Region*				
Western	70	2.12 (0.82)	3.81 (1.15)	2.45 (1.28)
Central	54	2.72 (1.92)	3.69 (1.32)	2.53 (1.40)
Easterm	124	2.91 (1.40)	3.61 (1.27)	2.97 (1.25)
* Island/mainland*				
Island	96	2.23 (0.87)	3.74 (1.27)	2.66 (1.19)
Mainland	152	2.91 (1.65)	3.65 (1.24)	2.78 (1.39)
Total	248	2.65 (1.44)	3.65 (1.25)	2.73 (1.32)
2010 (19 villages)				
* Region*				
Western	119	1.53 (1.01)	3.49 (1.39)	2.25 (1.48)
Central	70	1.62 (0.51)	3.82 (1.52)	2.67 (1.09)
Eastern	169	1.31 (0.78)	3.40 (1.71)	2.08 (1.38)
* Island/mainland*				
Island	148	1.49 (0.95)	3.60 (1.56)	2.12 (1.32)
Mainland	210	1.42 (0.34)	3.45 (1.60)	2.35 (1.41)
Total	358	1.45 (0.83)	3.51 (1.58)	2.25 (1.38)
2010 (31 villages)				
* Region*				
Western	119	1.53 (1.01)	3.49 (1.39)	2.25 (1.48)
Central	219	1.42 (0.74)	3.55 (1.69)	2.24 (1.33)
Easterm	130	1.29 (0.81)	3.16 (1.21)	2.32 (1.36)
*Island/mainland*				
Island	148	1.49 (0.95)	3.60 (1.56)	2.12 (1.32)
Mainland	320	1.38 (0.78)	3.35 (1.48)	2.33 (1.40)
Total	468	1.41 (0.84)	3.43 (1.51)	2.27 (1.38)

Numbers in parenthesis are SD.

A cluster analysis grouped the 31 villages into three areas, western region, central region and eastern region (Figure 4B). The best GLMM for the number of residents included two fixed variables, region and island/mainland, and two random variables, house and village (Additional file 5: Table S5). While the effect of region was statistically significant (*χ*^*2*^ = 7.56, df = 2, P = 0.023), island/mainland was not statistically significant (*χ*^*2*^ = 2.62, df = 1, P = 0.105). The western region had about 1.5 times the number of residents when compared with the other regions (Table 2). The best model for the number of bed nets did not include any fixed variables. Although the best model for the number of residents having slept under bed nets selected one fixed variable, region, this variable was not significant (*χ*^*2*^ = 5.39, df = 2, P = 0.067).

### Adult species compositions of 19 villages in 2008 and 2010

A total of 1238 *An. gambiae* s.l. adults were collected from the 19 villages in 2008 (Additional file 2: Table S2). Of these, 1117 were used for PCR species identification. PCR successfully amplified 1067 (95.5%) samples, identifying 721 (67.6%) of these as *An. arabiensis,* and 346 (32.4%) as *An. gambiae* s.s. In 2010, a total of 1712 *An. gambiae* s.l. were collected from the same 19 villages, and 1285 of these were used for PCR identification. PCR successfully amplified 1190 (92.6%) samples, of which 546 (45.9%) were *An. arabiensis*, and 644 (54.1%) were *An. gambiae* s.s.

The best model for the relative abundance of *An. arabiensis* in the 19 villages selected three fixed variables, geographical region, island/mainland and year, and two random variables, house and village (Additional file 6: Table S6). The effects of these fixed variables were statistically significant (region: *χ*^*2*^ = 15.04, df = 2, P < 0.001; island/mainland: *χ*^*2*^ = 5.86, df = 1, P = 0.016; year: *χ*^*2*^ = 72.48, df = 1, P < 0.001). The relative abundance of *An. arabiensis* was significantly higher in the island population when compared with the mainland population, and higher in the central region and eastern region when compared with the western region (Figure 4A). In 2010, the relative abundance of *An. arabiensis* was significantly lower than in 2008 (Figure 4B).

### Adult densities of 19 villages in 2008 and 2010

The best Poisson GLMM for *An. gambiae* s.l. density selected four fixed variables, the number of bed nets, the number of residents, geographical region, and year, and two random variables, house and village (Additional file 7: Table S7). The effects of each fixed variable except region were statistically significant (bed net: *χ*^*2*^ = 67.26, df = 1, P < 0.001; resident: *χ*^*2*^ = 128.25, df = 1, P < 0.001; region: *χ*^*2*^ = 5.09, df = 1, P = 0.078; year: *χ*^*2*^ = 8.71, df = 1, P = 0.003). Increasing bed nets by one decreased the average density of *An. gambiae* s.l. per house by 10%, and increasing residents by one increased it by 13% (Table 1). The density of *An. gambiae* s.l. in 2008 was 17% lower than 2010.

The best model for the density of *An. gambiae* s.s. selected five fixed variables, the number of bed nets, the number of residents, island/mainland, region, and year, and two random variables, house and village (Additional file 7: Table S7). While the effects of the number of bed nets, the number of residents and region were statistically significant (bed net: *χ*^*2*^ = 9.78, df = 1, P = 0.002; resident: *χ*^*2*^ = 58.64, df = 1, P < 0.001; region: *χ*^*2*^ = 12.00, df = 2, P = 0.002), the effects of island/mainland and year were not significant (island/mainland:*χ*^*2*^ = 2.41, df = 1, P = 0.121; year: *χ*^*2*^ = 2.55, df = 1, P = 0.111). Increasing bed nets by one decreased the average density of *An. gambiae* s.s. per house by 8%, and increasing residents by one increased it by 13% (Additional file 7: Table S7). It’s average density was 41% and 67% lower in the central and eastern regions when compared with the western region, respectively.

The best model for *An. arabiensis* selected the number of bed nets, the number of residents, island/mainland, region and year, and two random variables, house and village (Additional file 7: Table S7), these fixed variables were statistically significant (bed net: *χ*^*2*^ = 84.94, df = 1, P < 0.001; resident: *χ*^*2*^ = 69.61, df = 1, P < 0.001; island/mainland: *χ*^*2*^ = 4.21, df = 1, P = 0.040; region: *χ*^*2*^ = 4.21, df = 2, P = 0.040; year: *χ*^*2*^ = 42.25, df = 1, P < 0.001). Increasing bed nets by one decreased the average number of *An. arabiensis* by 13%, and increasing residents by one increased it by 13% (Table 1). Its density was 2 times higher in the mainland compared with the island region, and 4.7 times and 3.5 times higher in the central region and eastern region compared with the western region, respectively. In 2010, the density of *An. arebiensis was* 46% lower than 2008.

### Adult species composition of 31 villages in 2010

In 2010, 2017 *An. gambiae* s.l. were collected from the 31 villages, of these 1548 were used for PCR (Additional file 2: Table S2). The number of samples successfully identified was 1412 (91.2%). Of these, 735 (52.1%) and 677 (47.9%) were *An. arabiensis* and *An. gambiae* s.s., respectively. The best binomial GLMM for the relative abundance of *An. arabiensis* in the 31 villages selected two fixed variables, island/mainland and region, and two random variables, house and village (Additional file 8: Table S8), only region was statistically significant (island/mainland: *χ*^*2*^ = 2.98, df = 1, P = 0.084; region: *χ*^*2*^ = 14.65, df = 2, P < 0.001). The relative abundance of *An. arabiensis* was higher in the eastern regions than in the western and central regions (Figure 4B).

### Adult densities of 31 villages in 2010

The best Poisson GLMM for the density of *An. gambiae* s.l. in the 31 villages selected two fixed variables, region and the number of residents, and two random variables, house and village (Additional file 9: Table S9). Although the effect of region was not statistically significant (*χ*^*2*^ = 4.86, df = 2, P = 0.088), the number of residents was significant (*χ*^*2*^ = 210.23, df = 1, P < 0.001). Increasing the number of residents by one increased the density of *An. gambiae* s.l. by 12%, the densities in the central and eastern regions were 42% greater and 30% lower than in the western region, respectively (Table 1).

The best GLMM for *An. gambiae* s.s. also selected the number of residents and region, using the same two random variables (Additional file 9: Table S9). These fixed variables were statistically significant (resident: *χ*^*2*^ = 53.43, df = 1, P < 0.001; region: *χ*^*2*^ = 19.41, df = 2, P < 0.001). Increasing the number of residents by one increased the density of *An. gambiae* s.s. by 11%, and the densities in the central and eastern regions were 19% and 87% lower than in the western region, respectively (Table 1).

For *An. arabiensis*, three fixed variables were used in the best model, the number of bed nets, the number of residents and region, the two random variables remained unchanged (Additional file 9: Table S9). While the number of residents was statistically significant (resident: *χ*^*2*^ = 117.59, df = 1, P < 0.001), the number of bed nets and region were not significant (*χ*^*2*^ = 3.20, df = 1, P = 0.074; region: *χ*^*2*^ = 5.96, df = 2, P = 0.051). Increasing the number of residents by one increased the density of *An. arabiensis* by 5%, and the densities in the central and eastern regions were 3.3 times greater than in the western region, respectively (Table 1).

## Discussion

The relative abundance of indoor resting *An. arabiensis* females in the study area had increased since the late 1990s. Although the adult densities of both *An. gambiae s.s*. and *An. arabiensis* decreased significantly from the late 90s, the increase in relative abundance of *An. arabiensis* was due to a striking decrease in *An. gambiae* s.s. This phenomenon has been reported from several locations in Africa [12,13,16]. The previous studies claimed that this is attributed to an increase of ITN coverage, as ITNs selectively reduce the abundance of endophagic *An. gambiae* s.s. [8-10]. Although bed net information in the study area in the late 90s was not available, it is clear that the bed net coverage dramatically increased after the mass ITN distribution, which started in 2006 and targeted pregnant women and infants [35,36]. In fact, a negative relationship between the number of bed nets and the density of vectors was seen in the results of the retrospective analysis; however, this was not the case in the cross sectional survey of the 31 villages, as the bed net coverage was relatively even among them.

The analyses for larval species composition also confirmed a decadal change. As expected [24], the relative abundance of *An. arabiensis* larvae was greater than that of indoor resting *An. arabiensis* females. As PSC targets indoor resting mosquitoes, this sampling method overestimates the abundance of endophilic *An. gambiae* s.s. females [8-10]. Considering that *An. arabiensis* and *An. gambiae* s.s*.* are mostly sympatric in their larval stage [4-6], the results from the larval study reflect the species composition of the study area more accurately.

However, the decadal change in vector species composition varied geographically. The relative abundance of *An. arabiensis* did not increase significantly in five villages, Takawiri, Kibuogi, Ragwe, Wanyama and Mfangano, from the late 90s, with *An. gambiae* s.s*.* remaining dominant in these villages. Although the relative abundance of *An. arabiensis* increased significantly in Ngodhe in 2008, *An. gambiae* s.s. was still dominant. In Kibuogi, the relative abundance of *An. arabiensis* had actually decreased since the late 90s. These phenomena cannot be explained simply by bed net coverage, because the geographical variation of bed net coverage in the study area was not significant in 2008 and 2010, and the variables related to bed nets were not significantly associated with the relative abundance of *An. arabiensis* during these years. Similarly, the bed net related variables were not significantly associated with vector densities when the study area was extended to the 31 villages, which further suggests that other factors have more influence on species composition in this study area [2,7,22].

The dominancy of *An. gambiae* s.s. in the villages studied may be related to the relatively low availability of livestock. Since, except for Ragwe, all of these villages are on islands, the grazing area for livestock is limited, which may reduce the availability of livestock for zoophilic *An. arabiensis*[8-10], and this in turn reduces the number of this species. The results from the analyses for the vector species compositions and densities in 2008 and 2010 also support this notion. *Anopheles arabiensis* dominated in the mainland and in the eastern region of the extended study area. The livestock grazing areas in the mainland are extensive, and the landscape becomes flatter in the eastern region. Although Ragwe is in the mainland, the village is surrounded by hills, and the livestock grazing is limited. On the other hand, the greater relationship of *An. gambiae* s.s. density with the number of residents (or its anthropophilic behavior) suggests that the dominancy of *An. gambiae* s.s. is related to human populations [37]. Although the difference in the number of residents was not statistically significant, it was higher in the island villages.

In addition to the availability of grazing area, the culture associated with the local ethnic groups may influence the abundance of livestock in the study area, and eventually influence the local vector species composition. The traditional lifestyle of the Luo is closely associated with livestock, while the focus of the Suba, who mainly inhabit the islands and the western region, is cultivating crops rather than pasturing livestock. As small boats are the only available transportation to the islands, the limited accessibility may also keep the abundance of livestock low on the islands.

Climate is another plausible factor for explaining the spatial variation of species composition. *Anopheles gambiae* s.s. has a lower desiccation tolerance than *An. arabiensis*, and *An. gambiae* s.s*.* tends to dominate in wetter areas when compared with *An. arabiensis*[7,22,38]. The villages that had abundant *An. gambiae* s.s. may have higher humidity. Although a climate data set at a fine spatial scale is not available in the study area, the climate in the western region may be wetter than the eastern region. The residents in the western region often plant maize twice a year, but a two-crop system is not common in the eastern region, which suggests that the amount of rainfall is greater in the western region. The remaining patches of indigenous forests also suggest a wetter climate in the western region, and the forests further retain humidity in the area [39]. The lake water may also maintain higher humidity on the islands. In addition, physical barriers such as lake water and hills limit the movement of mosquitoes, and so mosquito populations may become more stable on islands and in the western region (in particular, Ragwe is isolated from the other areas by its lake and hills).

Another interesting phenomenon observed in this study was that the relative abundance of *An. arabiensis* was significantly lower in 2010 when compared with 2008. This was attributed to the significant reduction of *An. arabiensis* density in 2010, and a slight increase in *An. gambiae* s.s. density. Although the increase of *An. gambiae* s.s. was not statistically significant, overall *An. gambiae* s.l. significantly increased in 2010. The increase of vector population may be due to the decrease of bed nets in 2010 versus 2008. The decrease in bed net numbers reduces the selective pressure against *An. gambiae* s.s., in particular [3,12-16]. The development of biochemical and/or behavioral insecticide resistance may also account for the increase in *An. gambiae* s.s. [3,19,30,40-42]. In the study area, Kawada *et al.* reported that the *An. gambiae* s.s. had developed insecticide resistance related with a high frequency of knockdown resistance mutation, while *An. arabiensis* had a comparatively low frequency [30]. However, the low frequency of a knockdown resistance mutation does not fully explain the decline of *An. arabiensis* in 2010, as this species had acquired another type of pyrethorid insecticide resistance related to P450 by 2010 [30].

Otherwise, the decrease of *An. arabiensis* may simply be an annual variation. The long rainy season in 2008 was slightly drier than in 2010 due to the occurrence of La Niña Modoki [43,44], which may have increased the relative abundance of *An. arabiensis* in 2008. But the wetter rainy season in 2010 should not decrease the actual abundance of *An. arabiensis*, as its abundance has always been positively associated with rainfall [45-48]. Another possible reason for the decline in *An. arabiensis* is the development of behavioral resistance to ITNs [3,19]. Although *An. arabiensis* is generally exophagic, the *An. arabiensis* population in the study area may have further enhanced its exophagic behavior, which may have consequently reduced the number of indoor catches of this species using PSC. The larval species composition changed little between 2009 and 2010, which may support this explanation, although larvae were not sampled in the same year.

## Conclusions

*Anopheles gambiae* s.s. has been replaced by *An. arabiensis* as the dominant malaria vector species, and has experienced a reduction in density of nearly 95% in the study area over 10 years. These phenomena are most likely explained by the dramatic increase in ITN coverage [35]. However, this decadal replacement of dominant vector species was not seen in most island villages, and *An. gambiae* s.s. was still dominant in the western part of the study area. The dominance of *An. gambiae* s.s. is not simply explained by bed net coverage, but rather by other environmental factors such as livestock availability or climate. This study also observed a slight increase in malaria vectors and an increase in the relative abundance of *An. gambiae* s.s. in 2010 compared with 2008. The decrease of bed net coverage and development of insecticide resistance may partially explain these changes.

### Implication

As this study demonstrated that an increase in bed net coverage does not necessarily replace endophilic species with exophilic species, verifying the target vector species is important in vector control rather than assuming *An. arabiensis* as the primary target in a high bed net coverage area. An adequate vector control tool and duration of intervention should be carefully determined for a targeted area [49-52]. The increase in *An. gambiae* s.s. density in 2010 is alarming. Periodical monitoring of vector density, species composition and insecticide resistance is necessary to detect an unexpected resurgence in malaria vector population and parasite transmission [53-55]. Proper and regular maintenance of ITNs to ensure a good coverage is a minimum requirement to avoid such a resurgence [56].

## Abbreviations

AIC: Akaike Information Criterion; GLMM: Generalized linear mixed model; GPS: Global positioning system; IRS: Indoor residual spraying; ITN: Insecticide treated bed net; KEMRI: Kenya Medical Research Institute; PCR: rDNA-polymerase chain reaction; PSC: Pyrethrum spray catches.

## Competing interests

The authors declare that they have no competing interests.

## Authors’ contributions

KF and NM conceived and designed this study. SN helped in designing and planning the study in Kenya. KF, GD, GS and PA collected the field data, and KF, MM, SW, JL and JK organized and conducted the laboratory work. KF performed the data analyses. KF drafted the first manuscript, and KF and NM finalized the manuscript. All authors have read and approved the final manuscript.

## Supplementary Material

Additional file 1: Table S1 Results of the best binomial GLMM for the relative abundance of *An. arabiensis* larvae. The larvae were sampled in 1997, 2008 and 2010. The parameters for 2009 and 2010 were estimated based on 1997, and the parameters for area was estimated based on island. Click here for file

Additional file 2: Table S2 The numbers of *An. gambiae* s.l. females sampled from each villages in 1999, 2008 and 2010. Click here for file

Additional file 3: Table S3 Results of the best binomial GLMM for the relative abundance of *An. arabiensis* females in 11 villages. The mosquitoes were sampled in 1999, 2008 and 2010. The parameters for 2008 and 2010 were estimated based on 1999. Click here for file

Additional file 4: Table S4 Results of the best binomial GLMMs for the densities of malaria vectors in 11 villages. The mosquitoes were sampled in 1999, 2008 and 2010. Parameters for 2008 and 2010 were estimated based on 1999, and those for the eastern region was estimated based on the western region. Click here for file

Additional file 5: Table S5 Results of the best GLMMs for bed nets, residents and residents slept under nets. The parameters for mainland were estimated based on island, those for the central and eastern regions were estimated based on the western region, and those for 2010 were estimated based on 2008. Click here for file

Additional file 6: Table S6 Results of the best binomial GLMM for the relative abundance of *An. arabiensis* in 19 villages. The mosquitoes were sampled in 2009 and 2010. The parameters for mainland were estimated based on island, those for the central and eastern regions were estimated based on the western region, and those for 2010 were estimated based on 2008. Click here for file

Additional file 7: Table S7 Results of the best Poisson GLMM for the densities of malaria vectors in 19 villages. The mosquitoes were sampled in 2008 and 2010. The parameters for 2008 and 2010 were estimated based on 1999, and those for the eastern region was estimated based on the western region. Click here for file

Additional file 8: Table S8 Results of the best binomial GLMM for the relative abundance of *An. arabiensis* in 31 villages. The mosquitoes were sampled in 2008 and 2010. The mosquitoes were sampled in 2010. The parameters for 2008 and 2010 were estimated based on 1999, and those for the eastern region were estimated based on the western region. Click here for file

Additional file 9: Table S9 Results of the best Poisson GLMM for the densities of malaria vectors in 31 villages. The mosquitoes were sampled in 2010. The parameters for the central and eastern regions were estimated based on the western region. Click here for file
