# Supplementary material for: Impacts of insecticide treated bed nets on Anopheles gambiae s.l. populations in Mbita district and Suba district, Western Kenya
Source: Parasit Vectors. 2014 Feb 11;7:63. doi: 10.1186/1756-3305-7-63 (PMC3925958; doi:10.1186/1756-3305-7-63)
Supplement: Additional file 1: Table S1 — Results of the best binomial GLMM for the relative abundance of An. arabiensis larvae. The larvae were sampled in 1997, 2008 and 2010. The parameters for 2009 and 2010 were estimated based on 1997, and the parameters for area was estimated based on island. [file 1756-3305-7-63-S1.doc]

**Table S1.** **Results of the best binomial GLMM for the relative abundance of *An. arabiensis* larvae.** The larvae were sampled in 1997, 2008 and 2010. The parameters for 2009 and 2010 were estimated based on 1997, and the parameters for area was estimated based on island.

| Factors |  | Coefficients | SE | *Z* | P |
| --- | --- | --- | --- | --- | --- |
| (Intercept) |  | 2.18 | 0.875 | 2.49 | 0.013 |
| Year |  |  |  |  |  |
| 2009 |  | 2.33 | 0.234 | 9.98 | < 0.001 |
| 2010 |  | 5.28 | 0.722 | 7.31 | < 0.001 |
| Area (mainland) |  | -1.62 | 0.924 | -1.75 | 0.080 |
