# Supplementary material for: Impacts of insecticide treated bed nets on Anopheles gambiae s.l. populations in Mbita district and Suba district, Western Kenya
Source: Parasit Vectors. 2014 Feb 11;7:63. doi: 10.1186/1756-3305-7-63 (PMC3925958; doi:10.1186/1756-3305-7-63)
Supplement: Additional file 2: Table S2 — The numbers of An. gambiae s.l. females sampled from each villages in 1999, 2008 and 2010. [file 1756-3305-7-63-S2.docx]

**Table S2. The numbers of *An. gambiae* s.l. females sampled from each villages in 1999, 2008 and 2010.**

|  |  |  | No. of collected mosquitoes (No. of houses) | | |
| --- | --- | --- | --- | --- | --- |
| Villages | Ab.^a^ | Area | 1999 | 2008 | 2010 |
| Kibuogi | KB | Island | 272 (8) | 47 (12) | 59 (30) |
| Mfangano | MF | Island | 246 (5) | 45 (10) | 40 (20) |
| Ngodhe | NG | Island | 174 (28) | 73 (20) | 224 (30) |
| Takawiri | TK | Island | 122 (27) | 109 (30) | 43 (30) |
| Kamsengre | KM | Island | 261 (4) | 54 (10) | 119 (10) |
| Utajo | UT | Island | 213 (4) | 37 (10) | 35 (20) |
| Wanyama | WN | Island | 288 (4) | 18 (10) | 95 (10) |
| Gingo | GN | Main | 209 (5) | 41 (10) | 83 (10) |
| Mbita | MB | Main | 1451 (50) | 35 (40) | 32 (40) |
| Ragwe | RG | Main | 610 (4) | 72 (10) | 61 (20) |
| Roo | RO | Main | 253 (5) | 25 (10) | 74 (20) |
| Kaugege | KG | Main | NA | 61 (10) | 406 (20) |
| Kirindo | KR | Main | NA | 26 (10) | 85 (10) |
| Kisui | KS | Main | NA | 64 (10) | 72 (20) |
| Luanda | LU | Main | NA | 85 (10) | 49 (10) |
| Nyamanga | NM | Main | NA | 175 (20) | 74 (30) |
| Tabla | TB | Main | NA | 220 (10) | 129 (10) |
| Waturi | WT | Main | NA | 6 (10) | 12 (10) |
| Nyaroya | NR | Main | NA | 45 (5) | 60 (10) |
| Akuot | AK | Main | NA | NA | 7 (10) |
| Alala | ALA | Main | NA | NA | 27 (5) |
| Alero | ALE | Main | NA | NA | 11 (5) |
| Gode Ariyo | GA | Main | NA | NA | 17 (10) |
| Kamsama | KMS | Main | NA | NA | 2 (10) |
| Kitare | KT | Main | NA | NA | 9 (10) |
| Mirunda | MR | Main | NA | NA | 16 (10) |
| Misori | MS | Main | NA | NA | 15 (10) |
| Ng'ou | NU | Main | NA | NA | 29 (10) |
| Nyandago | ND | Main | NA | NA | 87 (10) |
| Obambo | OB | Main | NA | NA | 36 (10) |
| Uwi | UW | Main | NA | NA | 9 (10) |
| Total |  |  | 4099 (144) | 1238 (255) | 2017 (470) |

^a^ Abbreviation of village name.
