# Supplementary material for: Impacts of insecticide treated bed nets on Anopheles gambiae s.l. populations in Mbita district and Suba district, Western Kenya
Source: Parasit Vectors. 2014 Feb 11;7:63. doi: 10.1186/1756-3305-7-63 (PMC3925958; doi:10.1186/1756-3305-7-63)
Supplement: Additional file 3: Table S3 — Results of the best binomial GLMM for the relative abundance of An. arabiensis females in 11 villages. The mosquitoes were sampled in 1999, 2008 and 2010. The parameters for 2008 and 2010 were estimated based on 1999. [file 1756-3305-7-63-S3.docx]

**Table S3. Results of the best binomial GLMM for the relative abundance of *An. arabiensis* females in 11 villages.** The mosquitoes were sampled in 1999, 2008 and 2010. The parameters for 2008 and 2010 were estimated based on 1999.

| Factors |  | Coefficients | SE | *Z* | P |
| --- | --- | --- | --- | --- | --- |
| (Intercept) |  | -2.53 | 0.333 | -7.60 | < 0.001 |
| Year |  |  |  |  |  |
| 2008 |  | 2.41 | 0.209 | 11.53 | < 0.001 |
| 2010 |  | 1.35 | 0.202 | 6.67 | < 0.001 |
