# Supplementary material for: Impacts of insecticide treated bed nets on Anopheles gambiae s.l. populations in Mbita district and Suba district, Western Kenya
Source: Parasit Vectors. 2014 Feb 11;7:63. doi: 10.1186/1756-3305-7-63 (PMC3925958; doi:10.1186/1756-3305-7-63)
Supplement: Additional file 5: Table S5 — Results of the best GLMMs for bed nets, residents and residents slept under nets. The parameters for mainland were estimated based on island, those for the central and eastern regions were estimated based on the western region, and those for 2010 were estimated based on 2008. [file 1756-3305-7-63-S5.docx]

**Table S5. Results of the best GLMMs for bed nets, residents and residents slept under nets**. The parameters for mainland were estimated based on island, those for the central and eastern regions were estimated based on the western region, and those for 2010 were estimated based on 2008.

| Factors |  | Coefficient | SE | z | P |
| --- | --- | --- | --- | --- | --- |
| A. 19 villages in 2008 &2010 |  |  |  |  |  |
| Bed net |  |  |  |  |  |
| (Intercept) |  | 1.32 | 0.140 | 9.39 | < 0.001 |
| Island/mainland |  |  |  |  |  |
| Mainland |  | 0.26 | 0.175 | 1.48 | 0.139 |
| Year |  |  |  |  |  |
| 2010 |  | -0.73 | 0.061 | -12.01 | < 0.001 |
| Resident |  |  |  |  |  |
| (Intercept) |  | 1.63 | 0.100 | 16.31 | < 0.001 |
| Region |  |  |  |  |  |
| Central |  | 0.32 | 0.139 | 2.33 | 0.020 |
| Eastern |  | 0.25 | 0.121 | 2.08 | 0.038 |
| Year |  |  |  |  |  |
| 2010 |  | -0.10 | 0.045 | -2.21 | 0.027 |
| Resident under net |  |  |  |  |  |
| (Intercept) |  | 1.28 | 0.133 | 9.63 | < 0.001 |
| Region |  |  |  |  |  |
| Central |  | 0.30 | 0.187 | 1.61 | 0.107 |
| Eastern |  | 0.34 | 0.162 | 2.12 | 0.034 |
| Year |  |  |  |  |  |
| 2010 |  | -0.26 | 0.054 | -4.82 | < 0.001 |
| B. 31 villages in 2010 |  |  |  |  |  |
| Bed net |  |  |  |  |  |
| (Intercept) |  | 0.93 | 0.123 | 7.51 | < 0.001 |
| Island/mainland |  |  |  |  |  |
| Mainland |  | -0.09 | 0.144 | -0.62 | 0.538 |
| Resident |  |  |  |  |  |
| (Intercept) |  | 1.60 | 0.133 | 12.08 | < 0.001 |
| Island/mainland |  |  |  |  |  |
| Mainland |  | -0.23 | 0.141 | -1.66 | 0.098 |
| Region |  |  |  |  |  |
| Central |  | 0.43 | 0.150 | 2.83 | 0.005 |
| Eastern |  | 0.43 | 0.169 | 2.52 | 0.012 |
| Resident under net |  |  |  |  |  |
| (Intercept) |  | 1.09 | 0.134 | 8.08 | < 0.001 |
| Region |  |  |  |  |  |
| Central |  | 0.33 | 0.163 | 2.00 | 0.045 |
| Eastern |  | 0.39 | 0.164 | 2.38 | 0.017 |
