# Supplementary material for: Impacts of insecticide treated bed nets on Anopheles gambiae s.l. populations in Mbita district and Suba district, Western Kenya
Source: Parasit Vectors. 2014 Feb 11;7:63. doi: 10.1186/1756-3305-7-63 (PMC3925958; doi:10.1186/1756-3305-7-63)
Supplement: Additional file 6: Table S6 — Results of the best binomial GLMM for the relative abundance of An. arabiensis in 19 villages. The mosquitoes were sampled in 2009 and 2010. The parameters for mainland were estimated based on island, those for the central and eastern regions were estimated based on the western region, and those for 2010 were estimated based on 2008. [file 1756-3305-7-63-S6.docx]

**Table S6. Results of the best binomial GLMM for the relative abundance of *An. arabiensis* in 19 villages.** The mosquitoes were sampled in 2009 and 2010. The parameters for mainland were estimated based on island, those for the central and eastern regions were estimated based on the western region, and those for 2010 were estimated based on 2008.

| Factors |  | Coefficient | SE | *Z* | P |
| --- | --- | --- | --- | --- | --- |
| (Intercept) |  | -1.37 | 0.531 | -2.58 | 0.010 |
| Island/mainland |  |  |  |  |  |
| Mainland |  | 1.43 | 0.537 | 2.66 | 0.008 |
| Region |  |  |  |  |  |
| Central |  | 1.18 | 0.681 | 1.73 | 0.084 |
| Eastern |  | 2.90 | 0.639 | 4.55 | < 0.001 |
| Year |  |  |  |  |  |
| 2010 |  | -1.38 | 0.165 | -8.36 | < 0.001 |
