# Supplementary material for: Impacts of insecticide treated bed nets on Anopheles gambiae s.l. populations in Mbita district and Suba district, Western Kenya
Source: Parasit Vectors. 2014 Feb 11;7:63. doi: 10.1186/1756-3305-7-63 (PMC3925958; doi:10.1186/1756-3305-7-63)
Supplement: Additional file 7: Table S7 — Results of the best Poisson GLMM for the densities of malaria vectors in 19 villages. The mosquitoes were sampled in 2008 and 2010. The parameters for 2008 and 2010 were estimated based on 1999, and those for the eastern region was estimated based on the western region. [file 1756-3305-7-63-S7.doc]

**Table S7. Results of the best Poisson GLMM for the densities of malaria vectors in 19 villages.** The mosquitoes were sampled in 2008 and 2010. The parameters for 2008 and 2010 were estimated based on 1999, and those for the eastern region was estimated based on the western region.

| Factors |  | Coefficients | SE | *Z* | *P* |
| --- | --- | --- | --- | --- | --- |
| *An. gambiae* s.l. |  |  |  |  |  |
| (Intercept) |  | 0.35 | 0.245 | 1.42 | 0.156 |
| Bed net |  | -0.10 | 0.014 | -7.73 | < 0.001 |
| Resident |  | 0.12 | 0.011 | 11.13 | < 0.001 |
| Region |  |  |  |  |  |
| Central |  | 0.78 | 0.342 | 2.28 | 0.023 |
| Eastern |  | 0.17 | 0.299 | 0.58 | 0.562 |
| Years |  |  |  |  |  |
| 2010 |  | -0.19 | 0.064 | -2.95 | 0.003 |
| *An. gambiae* s.s. |  |  |  |  |  |
| (Intercept) |  | -0.04 | 0.369 | -0.12 | 0.907 |
| Bed net |  | -0.08 | 0.025 | -3.02 | 0.003 |
| Resident |  | 0.12 | 0.016 | 7.75 | < 0.001 |
| Island/Mainland |  |  |  |  |  |
| Mainland |  | -0.60 | 0.377 | -1.60 | 0.110 |
| Region |  |  |  |  |  |
| Central |  | 0.53 | 0.476 | 1.10 | 0.270 |
| Eastern |  | -1.12 | 0.445 | -2.51 | 0.012 |
| Years |  |  |  |  |  |
| 2010 |  | 0.14 | 0.088 | 1.59 | 0.111 |
| *An. arabiensis* |  |  |  |  |  |
| (Intercept) |  | -1.30 | 0.351 | -3.72 | < 0.001 |
| Bed net |  | -0.14 | 0.017 | -8.45 | < 0.001 |
| Resident |  | 0.12 | 0.014 | 8.25 | < 0.001 |
| Island/Mainland |  |  |  |  |  |
| Mainland |  | 0.74 | 0.335 | 2.20 | 0.028 |
| Region |  |  |  |  |  |
| Central |  | 1.54 | 0.436 | 3.53 | < 0.001 |
| Eastern |  | 1.29 | 0.396 | 3.25 | 0.001 |
| Year |  |  |  |  |  |
| 2010 |  | -0.61 | 0.096 | -6.37 | < 0.001 |
