# Supplementary material for: Impacts of insecticide treated bed nets on Anopheles gambiae s.l. populations in Mbita district and Suba district, Western Kenya
Source: Parasit Vectors. 2014 Feb 11;7:63. doi: 10.1186/1756-3305-7-63 (PMC3925958; doi:10.1186/1756-3305-7-63)
Supplement: Additional file 8: Table S8 — Results of the best binomial GLMM for the relative abundance of An. arabiensis in 31 villages. The mosquitoes were sampled in 2008 and 2010. The mosquitoes were sampled in 2010. The parameters for 2008 and 2010 were estimated based on 1999, and those for the eastern region were estimated based on the western region. [file 1756-3305-7-63-S8.doc]

**Table S8. Results of the best binomial GLMM for the relative abundance of *An. arabiensis* in 31 villages.** The mosquitoes were sampled in 2008 and 2010. The mosquitoes were sampled in 2010. The parameters for 2008 and 2010 were estimated based on 1999, and those for the eastern region was estimated based on the western region.

| Factors |  | Coefficient | SE | *Z* | P |
| --- | --- | --- | --- | --- | --- |
| (Intercept) |  | -1.97 | 0.576 | -3.41 | < 0.001 |
| Island/mainland |  |  |  |  |  |
| Mainland |  | 1.03 | 0.575 | 1.80 | 0.073 |
| Region |  |  |  |  |  |
| Central |  | 1.34 | 0.626 | 2.15 | 0.032 |
| Eastern |  | 2.99 | 0.732 | 4.08 | < 0.001 |
