# Supplementary material for: Impacts of insecticide treated bed nets on Anopheles gambiae s.l. populations in Mbita district and Suba district, Western Kenya
Source: Parasit Vectors. 2014 Feb 11;7:63. doi: 10.1186/1756-3305-7-63 (PMC3925958; doi:10.1186/1756-3305-7-63)
Supplement: Additional file 9: Table S9 — Results of the best Poisson GLMM for the densities of malaria vectors in 31 villages. The mosquitoes were sampled in 2010. The parameters for the central and eastern regions were estimated based on the western region. [file 1756-3305-7-63-S9.doc]

**Table S9. Results of the best Poisson GLMM for the densities of malaria vectors in 31 villages.** The mosquitoes were sampled in 2010. The parameters for the central and eastern regions were estimated based on the western region.

| Factors |  | Coefficient | SE | *Z* | P |
| --- | --- | --- | --- | --- | --- |
| *An. gambiae* s.l. |  |  |  |  |  |
| (Intercept) |  | 0.19 | 0.344 | 0.55 | 0.584 |
| Resident |  | 0.12 | 0.007 | 15.87 | < 0.001 |
| Region |  |  |  |  |  |
| Central |  | 0.35 | 0.408 | 0.87 | 0.386 |
| Eastern |  | -0.36 | 0.407 | -0.88 | 0.380 |
| *An. gambiae* s.s. |  |  |  |  |  |
| (Intercept) |  | -0.06 | 0.415 | -0.15 | 0.885 |
| Resident |  | 0.11 | 0.014 | 7.63 | < 0.001 |
| Region |  |  |  |  |  |
| Central |  | -0.21 | 0.494 | -0.44 | 0.663 |
| Eastern |  | -2.08 | 0.533 | -3.91 | < 0.001 |
| *An. arabiensis* |  |  |  |  |  |
| (Intercept) |  | -1.68 | 0.437 | -3.85 | < 0.001 |
| Bed net |  | 0.05 | 0.029 | 1.81 | 0.070 |
| Resident |  | 0.11 | 0.009 | 12.15 | < 0.001 |
| Region |  |  |  |  |  |
| Central |  | 1.19 | 0.507 | 2.34 | 0.019 |
| Eastern |  | 1.20 | 0.502 | 2.40 | 0.016 |
